# Do wearable alcohol-based handrub dispensers increase hand hygiene compliance? - a mixed-methods study

**DOI:** 10.1186/s13756-018-0439-5

**Published:** 2018-11-23

**Authors:** Jonas Keller, Aline Wolfensberger, Lauren Clack, Stefan P. Kuster, Mesida Dunic, Doris Eis, Yvonne Flammer, Dagmar I. Keller, Hugo Sax

**Affiliations:** 1 Division of Infectious Diseases and Hospital Epidemiology, University Hospital Zurich, University of Zurich, Rämistrasse 100, CH-8091 Zurich, Switzerland; 2 Emergency Department, University Hospital Zurich, University of Zurich, Zurich, Switzerland; 3 Baraka Health Centre, German Doctors Nairobi, Nairobi, Kenya

**Keywords:** Hand hygiene, Compliance, Wearable dispensers, Pocket dispensers, Clip on dispensers, Emergency room, Availability, Point of care

## Abstract

**Background:**

Hand Hygiene (HH) compliance was shown to be poor in several studies. Improving the availability of alcohol-based hand rub (ABHR) is a cornerstone for increasing HH compliance.

**Methods:**

In this study, we introduced wearable dispensers for ABHR in an Emergency Department (ED) well equipped with mounted ABHR dispensers and accompanied this single-modal intervention by a quasi-experimental mixed-method study. The study was performed in the ED of the University Hospital Zurich, Switzerland, a 950-bed tertiary teaching hospital. During a five-week baseline period and a seven-week intervention period, we observed HH compliance according to the WHO ‘Five Moments’ concept, measured ABHR consumption, and investigated perceived ABHR availability, self-reported HH compliance and knowledge of HH indications by questionnaire. Multivariable logistic regression was used to identify independent determinants for HH compliance. In addition, semi-structured interviews were conducted and thematically analyzed to assess barriers and facilitators for the use of the newly introduced dispensers.

**Results:**

Across 811 observed HH opportunities, the HH compliance for all moments was 56% (95% confidence interval (CI), 51–62%) during baseline and 64% (CI, 59–68%) during intervention period, respectively. In the multivariable analysis adjusted for sex, profession, and WHO HH moment, there was no difference in HH compliance between baseline and intervention (adjusted Odds ratio: 1.22 (0.89–1.66), *p* = 0.22), No significant changes were observed in consumption and perceived availability of ABHR. During intervention, 7.5% ABHR was consumed using wearable dispensers. HCP perceived wearable dispensers as unnecessary since mounted dispensers were readily accessible. Poor ergonomic design of the wearable dispenser emerged as a main barrier, especially its lid and fastening mechanism. Interviewees identified two ideal situations for wearable dispensers, HCP who accompany patients from ED to other wards, and HCP approaching a patient from a non-patient areas in the ED such as the central working station or the meeting room.

**Conclusion:**

The introduction of wearable dispensers did not increase observed hand hygiene compliance or ABHR consumption in an ED already well equipped with mounted dispensers. For broader acceptance and use, wearable dispensers might benefit from an optimized ergonomic design.

## Background

Despite significant advances in infection control, healthcare-associated infections represent a major challenge to modern medicine [1]. Appropriate hand hygiene (HH) is considered crucial to reduce the transmission of nosocomial pathogens and helps to prevent hospital-acquired infections [2, 3]. However, HH compliance has been shown to be poor in a multitude of studies [4, 5]. Data about HH compliance in Emergency Departments (ED) is limited, and results vary greatly [6]. ED physicians had a higher risk for non-adherence to HH compared to physicians working in other departments [7]. The mean rate of HH indications is higher in the ED than in medical or surgical wards [8], and high workload has been identified as a risk factor for low HH compliance [5].

To improve HH compliance, WHO names two strategies with known effectiveness: introducing widely accessible alcohol based hand rub (ABHR), and multifaceted interventions [4]. Again, in multifaceted approaches, improving the availability of ABHR is often an element of success [7]. Providing ABHR at the ‘point of care’ – requiring ABHR to be easily accessible and as close as possible (e.g. within arm’s reach), where patient care or treatment is taking place – is a cornerstone of the WHO strategy to improve HH compliance [4]. Accessibility can be achieved by introducing wearable dispensers with ABHR [9, 10]. But, in a single intervention study in 2008, Haas et al. showed that availability of wearable ABHR dispensers alone was not associated with a significant improvement in HH compliance in an ED [11].

For many years, ED personnel in our hospital expressed the wish to benefit from wearable dispensers. All wards of the University Hospital Zurich (UHZ), including the ED, are well equipped with mounted ABHR dispensers in close vicinity of patients, and up to this study, wearable dispensers were only available for staff in a few selected wards, e.g. in neonatology. In spring 2016, the infection prevention and control (IPC) team decided to meet the ED’s wish for wearable dispensers. The introduction was accompanied by a mixed-method study to investigate HH compliance, ABHR consumption, and the attitude of healthcare providers (HCP) towards wearable dispensers.

## Methods

### Study setting and population

The study was conducted between April and June 2016 at the ED of the UHZ, Switzerland. The UHZ is a 950-bed tertiary hospital covering all medical specialties except pediatrics and orthopedics. The ED holds 17 beds, and a mean of 44′000 patients are admitted to the ED yearly. The ED staff consists of 100 nurses, 13 staff emergency physicians, 25 medical interns, and various other professions (e.g. maintenance and nursing assistants). Non-ED consulting physicians and surgeons regularly visiting the ED. The study population for HH observation and interviews consisted of ED staff only, ABHR consumption was measured overall, thus including also HCP visiting the ER. The ED has an open floor plan with a central nursing and working station, and single patient bays separated by privacy curtains. Mounted dispensers are available in or immediately outside every patient room/bay. In total, there are 34 wall-mounted or table-top dispensers available in close proximity, i.e. below 2 m, of each patient bed. Additional dispensers exist in the staff lounge, storage rooms, and restrooms.

### Study design and endpoints

This mixed-method quasi-experimental before-after study evaluated the effect of introducing wearable ABHR dispensers, i.e. the intervention, by multiple measures. The main outcome was the observed HH compliance, defined as the proportion of individual HH indications met by a HH action [12]. Secondary outcomes were the measured consumption of ABHR and self-reported HH compliance. In addition, we investigated the perception and attitude of ED HCP regarding wearable dispensers by self-applied questionnaires and semi-structured interviews.

### Intervention

The study consisted of a five-week baseline period and a seven-week intervention period that started with the introduction of wearable dispensers. The wearable dispenser (B Braun®, 100 ml, dimensions 7x8x2.2 cm) included clips to attach the dispensers to the hospital apparel. Promotion of the wearable dispensers occurred in several staff-meetings, by user information via email, and by distribution of dispensers to HCP in person. The number and position of the mounted dispensers remained unchanged.

### Effect evaluation

HH compliance, the main outcome parameter, was assessed according to the WHO ‘My 5 moments for hand hygiene’ observation method through direct observation by two trained and validated members of the IPC team (MD and JK) [13, 14]. They recorded HH opportunities, HH actions, and HCP profession and sex in an anonymized form. During the last three weeks of the intervention period, the usage of either wearable dispensers or mounted dispensers was additionally noted during observations. Observations were done during weekdays at different time points between 8 am and 6 pm. Consumption of ABHR from mounted dispensers and wearable dispensers was assessed by weekly weighing the dispensers and counting the stock to calculate consumption.

Perceived ABHR availability, self-reported HH compliance, and knowledge of the WHO ‘My five moments for HH’ was assessed through a self-administered 3-item questionnaire using a 5-item Likert scale (Table 1). Additionally, participants indicated their profession and sex. Questionnaires were distributed to physicians’ physical mailboxes and were made available in the nurses’ common room after the baseline period and at the end of the intervention period.

**Table 1:** Questionnaire for self-evaluation of participants

No.	Question	Likert-Scale
1.	Have you memorized the “WHO five moments for hand hygiene” so that you utilize them automatically in daily routine?	1 = no
		2 = some of them
		3 = many of them
		4 = most of them
		5 = yes, completely
2.	How often do you correctly conduct hand hygiene according to the “WHO five moments for hand hygiene”?	1 = never
		2 = sometimes
		3 = often
		4 = most of the time
		5 = always
3.	Is ABHR sufficiently available to conduct hand hygiene according to the “WHO five moments for hand hygiene”?	1 = never
		2 = sometimes
		3 = often
		4 = most of the time
		5 = always

Abbreviations: *ABHR* alcohol based hand rub, *No.* Number, *WHO* World Health Organisation

### Sample size calculation and statistical analysis

The needed number of HH opportunities in baseline and intervention was calculated on the assumption that HH at baseline would be 50%, based on earlier observations using the same observation method. A clinically meaningful increase in HH compliance was judged to be at least 15 percentage points, i.e. from 50 to 65%. Requiring equal sample sizes for baseline and intervention, and assuming an average cluster size of three, a two-sided alpha of 0.05, and a power of 80% resulted in twice 339 opportunities.

Descriptive statistics were used to summarize data on HH compliance, ABHR consumption, and results of the questionnaire. Chi-square test was used to test differences in categorical variables. For comparison of continuous variables we used students t-test or anova, as appropriate. Multivariable logistic regression was used to adjust the effect of the intervention on HH compliance for potential confounders. We included the variables intervention period, sex, profession, hand hygiene moment and their respective subcategories in the multivariable model. We applied a chi-square test for trend to evaluate the change in consumption of ABHR from wearable dispensers as a proportion of overall ABHR consumption over time (‘ptest’, trend analysis for proportions). A *p*-value of <.05 was considered statistically significant. All statistical analysis were performed with STATA version 15 (Stata Corp., College Station, TX, USA).

### Semi-structured interviews and qualitative analysis

Semi-structured interviews were conducted with a convenience sample of nurses and physicians working in the ED at the end of the intervention period. The interview guide is displayed in Table 2. All interviews were audio-taped after obtaining oral informed consent and transcribed verbatim. Data analysis of anonymized interviews was conducted inductively, following a grounded theory approach [15]. Two researchers (JK and AW) independently read transcripts and identified emerging themes. Emerging themes were then discussed and final categories were established through consensus. Quotes about barriers and facilitators were semi-quantified by a scoring system: three points for quotes mentioned by a majority of participants, two points for quotes mentioned by a minority but two or more, one point for quotes mentioned by a single participant.

**Table 2:** Interview guide for the semi-structured interviews

No.	Question
1.	What do you think of the recently introduced wearable dispensers?
2.	How frequently have you used the wearable dispensers?
3.	What are the advantages/disadvantages of the wearable dispensers?
4.	Are there any situations where you prefer using either the wearable dispensers or the mounted dispensers?
5.	Do you have ideas to improve the wearable dispensers?

## Results

### Hand hygiene compliance

**Fig. 1:**
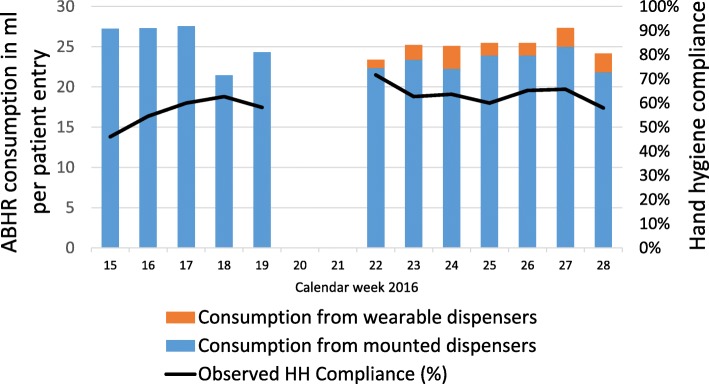
Hand hygiene compliance and consumption of alcohol based hand rub. Legend: The bar graph depicts consumption of ABHR in milliliters by mounted dispensers (blue bars) and wearable dispensers (orange bars). The line graph depicts HH compliance in percent. The five-week baseline period (weeks 15 to 19) and the seven-week intervention period (weeks 22 to 28) are separated by a two-week (weeks 20 and 21) period dedicated to preparing the intervention. Abbreviations: ABHR = Alcohol-based hand rub; HH = hand hygiene; ml = milliliters

A total of 811 HH opportunities were observed, 328 (40.4%) in the baseline period and 483 (59.6%) in the intervention period. The average HH compliance rose from 56% during baseline to 64% during intervention with a univariable odds ratio of 1.36 (1.02–1.81; *p* = .035). When adjusted for HH moment, sex and profession in the multivariable logistic regression model, odds ratio was 1.22 (0.89–1.66; *p* = .218) (Table 3). Weekly compliance across all indications are displayed in Fig. 1. In intervention, 7.3% (95% confidence interval (CI), 3.6–13.0) of observed HH actions were performed using wearable dispensers.

**Table 3:** Univariable and multivariable analysis of predictors for hand hygiene compliance

	Baseline period		Intervention period		Univariable analysis	Multivariable analysis
	HH opportunities	HH actions (%)	HH opportunities	HH actions (%)	Odds ratio (CI95%)	Odds ratio (CI95%)
Intervention period (vs. baseline period)	328	185 (56)	483	308 (64)	1.36 (1.02–1.81)	1.22 (0.89–1.66)
WHO HH moment						
Before touching patient	87	31 (36)	106	39 (37)	1	1
Before aseptic procedure	21	12 (57)	47	37 (79)	4.53 (2.47–8.30)	3.10 (1.66–5.79)
After body fluid exposure risk	38	27 (71)	59	47 (80)	5.65 (3.25–9.82)	4.49 (2.54–7.95)
After touching patient	162	109 (67)	254	180 (71)	4.00 (2.79–5.73)	4.05 (2.80–5.86)
After touching patient surroundings	20	6 (30)	17	5 (29)	0.74 (0.35–1.60)	0.61 (0.28–1.35)
Sex						
Female	206	125 (61)	360	245 (68)	1	1
Male	122	60 (49)	123	63 (51)	0.53 (0.39–0.72)	0.72 (0.50–1.02)
Profession						
Physician	114	57 (50)	156	74 (47)	1	1
Nurse	210	128 (61)	313	224 (72)	2.18 (1.62–2.95)	1.92 (1.34–2.76)
Other	4	0 (0)	14	10 (71)	1.33 (0.51–3.46)	0.93 (0.34–2.51)

Abbreviations: *CI* confidence interval, *n.a*. not applicable, *HH* hand hygiene

### Consumption of alcohol-based hand rub

Figure 1 shows the weekly consumption of ABHR, which was 25.6 ml per patient admission during baseline, and 25.2 ml per patient admission during intervention. During the intervention period, 7.5% of the total ABHR consumption resulted from use of wearable dispensers, confirming the abovementioned 7.3% of observed use. Consumption of ABHR from wearable dispensers did increase over the seven-week intervention period (*p* < .001).

### Perceived ABHR availability, self-reported hand hygiene compliance, and knowledge of HH moments

A total of 87 questionnaires were returned, 51 during baseline (20 (39%) by physicians, 27 (53%) by nurses, and 4 (8%) by others), and 36 during intervention (12 (33%) by physicians, 22 (61%) by nurses, and 2 (6%) by others).

The perceived availability of ABHR did not increase from baseline to intervention (4.47 vs 4.64, *p* = .31). Self-reported HH compliance did not differ between baseline and intervention (4.12 vs 4.03, *p* = .56), neither between sex (female vs male, 4.14 vs 3.97, *p* = .28), nor profession (physician vs nurse vs other, 4.09 vs 4.08 vs 4.0, *p* = .96). Mean self-reported knowledge of HH indications did not differ between baseline and intervention (4.27 vs. 4.28, *p* = .98). Overall, women’s perception of their own knowledge was better than men’s (4.40 vs 4.03, *p* = .024), whereas profession had no impact on the self-reported knowledge (physician vs nurse vs others, 4.31 vs 4.29 vs 4.0, *p* = .61).

### Semi-structured interviews

Overall, 24 participants took part in the interviews, 14 (58%) nurses, nine physicians (38%), and one (4%) nursing assistant; six (25%) participants were male. The interviews had a mean duration of 10 min. Thirteen of the interviewees (54%) had a negative overall impression of the wearable dispensers, eight (33%) were neutral, and three (13%) were positive.

We identified four categories of barriers and facilitators for the use of the wearable dispensers: *usability* characteristics of the current wearable dispenser, *availability* of ABHR, *cues to action/cognition* (i.e. subconscious or conscious activation towards use), and perceptions about the *safety*of wearable dispensers. Selected typical interview quotes are listed in Table 4. A majority of facilitators was categorized to “availability”. HCPs liked the principal idea of carrying the hand rub on them and also mentioned certain situations in which the wearable dispenser was of use, e.g. heading towards a patient from an area without mounted dispensers, and accompanying patient to wards where location of mounted dispensers was not familiar. In addition, interviewees perceived the wearable dispenser as a reminder to perform hand hygiene. Barriers were more often mentioned than facilitators, and arguments from all thematic groups emerged. Concerning usability, interviewees did not like the way the wearable dispensers were attached to the uniform because it made the dispenser dangle back and forth, and the fastening mechanism often failed, dropping the dispenser. HCP perceived the wearable dispensers as an additional item to carry around, while they already carried a multitude of other items such as stethoscopes and notes in their pockets, especially when wearing scrubs without coat. Wearable dispensers were mainly perceived to be unnecessary, given that a mounted dispenser was in easy reach during the vast majority of HH indications. HCPs were used to the mounted dispensers and habitually used them instead of the wearable dispensers. The outer surface of the wearable dispensers was perceived as risk factor for contaminating patients and hospital surroundings.

**Table 4:** Typical quotes about barriers and facilitators for use of wearable dispensers

	Facilitators	Barriers
Usability	Fastening mechanism ●○○	Fastening mechanism ●●●
	- “I generally like the possibility to attach the wearable dispensers to one’s trousers.”	- “During a CT scan of a patient I accompanied, the wearable dispenser came off four times.”
	- Dimensions ●○○	- “The wearable dispenser dangles constantly, and its weight pulls on my trousers which is really uncomfortable.”
	- “The size and weight of the wearable dispenser is perfect. If it was bigger, it couldn’t be put it in my pockets anymore.”	
		Opening and closing mechanism ●●○
		- “It is difficult to close the bottle with one hand only.”
		- Dosing of ABHR ●○○
		- “It is more difficult to dose the appropriate amount of ABHR compared to wall-mounted dispensers”
		- Dimensions ●○○
		- “The wearable dispenser is so small that you have to exchange it too often.”
		Burden ●●●
		- “The wearable dispenser is just something more to carry around, and I have to carry many other things with me already.”
Availability	General ●●●	General ●●●
	- “I generally like the idea that I carry the ABHR with me and have it available all the time.”	- “No, I do not see any advantage of the wearable dispenser. We do have enough ABHR available in the ER.”
	Specific situations ●●○	
	- “Sometimes the next wall-mounted dispenser is 10 m away, then the wearable dispenser is of use.”	
	- “I can use the wearable dispenser and do HH while heading to a patient.”	
	- “The wearable dispenser is of use, when I shift a patient to a ward and do not know the locations of ABHR dispensers.”	
Cues to action / Cognition	Habitualness ●○○	Habitualness ●●○
	- “Yes, I did use the wearable dispenser. I was used to wearable dispensers from the hospital I worked before.”	- “I’m so used to all the wall-mounted dispensers… [that I did not use the wearable dispenser].”
	Reminder ●●○	
	- “The wearable dispenser is a good reminder for HH.”	
Safety	n.a.	Dispensers perceived as risk factor for contamination ●●○
		- “The problem is that the bottle has to be opened with contaminated hands and afterwards closed with clean hands.”
		- “The dirty bottle in contact with my clean clothes all the time.”

Abbreviations: *ABHR* alcohol based hand rub, *CT* Computer tomography, *ER* emergency room, *HH* hand hygiene, *●●●* Mentioned by a majority of participants. ●●○ Mentioned by ≥2 participants. ●○○ Mentioned by one single participant

Interviewees made numerous suggestions about how to improve the wearable dispenser, including reducing its size to make it better fit in coat pockets, modifying the bottle outlet, e.g. by replacing it by a membrane, to avoid the need to open its cap, and improving the attaching mechanism.

## Discussion

The purpose of this study was to examine whether a single intervention approach of introducing wearable ABHR dispensers in addition to existing mounted dispensers would increase observed HH compliance and ABHR consumption in our ED. Additionally, we aimed to explain the findings by qualitative and quantitative data on HCP perceptions and attitude. We found that the intervention did not improve HH compliance and consumption of ABHR. Less than a tenth of ABHR was consumed using wearable dispensers. Two main ‘barriers’ for the acceptance of the wearable dispensers explained this unexpectedly low uptake, namely the habitual use and perceived sufficient access to mounted dispensers and the flawed ergonomic design of the wearable dispensers.

Increasing availability of ABHR is an often-mentioned facilitator for HH compliance and the “WHO Guidelines on Hand Hygiene in Health Care” declare ABHR availability as a prerequisite for good HH compliance [16–18]. Introducing wearable dispensers is one possibility to increase availability. Many researchers introduce wearable dispensers as part of multifaceted interventions. Yeung et al. found that the introduction of wearable dispensers with education led to increased HH compliance in long-term care facilities [10] and Koff et al. found improvement in HH compliance of anesthesia providers through the use of wearable dispensers with an audible alarm [19]. Only rarely introduction of wearable dispensers was studied as single intervention. Parks et al. were able to show that HH compliance of a regional anesthesia team increases when wearable dispensers are worn on person [20]. In contrast, the introduction of wearable dispensers in the ED of our hospital did not significantly increase HH compliance. Our results confirm the findings of Haas et al. in 2008, who did not find an improvement in ABHR consumption after introducing wearable dispensers in an ED [11].

Our ED is well equipped with mounted dispensers, making ABHR available within maximum 2 m distance from every patient bed. Interviewees mentioned the abundant availability of dispensers as one of the most important reasons for not using wearable dispensers and did not perceive an increase in availability of ABHR during intervention. Nevertheless, prior to this study, HCPs in the ED had repetitively been expressing the wish to get access to wearable dispensers. The low uptake in this investigation came therefore as a surprise. Interviewees did not comment on the ED’s anecdotal wish for wearable dispensers, but we hypothesize that it was voiced mainly by HCPs who were used to wearable dispensers from other hospitals or was triggered by rare events where ABHR was not accessible, corresponding to a classical reporting bias. Yet, interviewees identified two situations where ABHR was not easily accessible: first, when approaching a patient from a non-patient area in the ED such as the central working station or the meeting room and second, when accompanying patients to locations with unfamiliar localization of mounted dispensers.

Poor usability of the wearable dispensers was seen as a main barrier for application and several HCP interviewees expressed safety concerns as the outer surface of the dispensers was perceived to contaminate clean hands and patients surroundings. The design of a medical device affects compliance and behavior. Human factors engineering principles like ‘affordance’ (i.e. making use intuitive) and ‘minimizing physical effort’ (i.e. making adherence convenient) were found to improve adherence when applied in the development of medical devices [21, 22]. Good usability of medical products was shown to improve patient safety, e.g. by reducing errors using blood glucose meters and increase compliance using hand sanitizers [23, 24]. Properties of a medical product can act as ‘forcing functions’. These ‘forcing functions’ limit user errors by prohibiting or facilitating specific actions, and stand at the top of scale of the ‘hierarchy of intervention effectiveness’ - structural and technological interventions are more reliable in shaping people’s behavior than human based interventions such as training and education [25]. Therefore, the wearable dispensers might benefit from an optimized design to encourage use and dismantle safety concerns.

Our study has limitations. First, the intervention period might have been too short to change a year-long habit. Second, although we were not aware of any other infection prevention and control promotional activity in the ER during the study periods we cannot fully exclude a time dependent bias. Third, as we only provided wearable dispensers to the ED staff and not to healthcare providers ‘visiting’ the ER (e.g. consultation service), the percentage of consumption from wearable dispensers might have been underestimated. Still, the amount of ABHR consumed by ‘visiting’ HCP is probably negligible. Forth, the Hawthorne effect and HH observation only during daytime, excluding night-shifts and weekends, might have skewed the HH observation results. Our results were, however, triangulated by ABHR consumption and the qualitative and quantitative investigation of perception and attitude of ED collaborators, which are strengths of the mixed-method study approach we used [26].

## Conclusion

In conclusion, we found that the a single intervention of introduction of wearable ADHR dispensers in a busy ED, that had already been well equipped with mounted ABHR dispensers, did not significantly improve hand hygiene compliance or ABHR consumption. The main barriers for their use according to HCPs were the competing benefit of well-placed and abundant mounted dispensers and the flawed ergonomic design.

## Abbreviations

ABHR: Alcohol-based hand rub
CI: Confidence interval
ED: Emergency Department
HCP: Healthcare provider
HH: Hand Hygiene
IPC: Infection prevention and control
UHZ: University Hospital Zurich
WHO: World health organization
